# Measurement protocols and intra-abdominal hypertension treatment

**DOI:** 10.1590/0100-6991e-20202838

**Published:** 2021-02-05

**Authors:** BRUNO M PEREIRA

**Affiliations:** 1 - World Society of the Abdominal Compartment, President (2017-2019) - Campinas - SP - Brasil; 2 - University of Vassouras, Pró-Reitoria de Pesquisa e Pós-Graduação - Vassouras - RJ - Brasil; 3 - Grupo Surgical, Cirurgia de Urgência e Emergência - Campinas - SP - Brasil

It was with great satisfaction that I read the article “Protocols for diagnosis and management of intra-abdominal hypertension in intensive care units”, published by Caldas and Ascenção^1^. Although there is still much work to be done regarding the diagnosis and treatment of intra-abdominal hypertension (IAH) and Abdominal Compartment Syndrome (ACS) at a national level, growing interest by surgeons is noted. In a quick PubMed database search, I found only four scientific articles with the descriptors “intra-abdominal hypertension” and “abdominal compartment syndrome” published in Brazilian journals. The good news, however, is that three of the four are concentrated in the past three years. Of course, other articles exist in different databases, but this number is still very small. The presented review work comes precisely to fill that gap of evidence in Portuguese idiom. However, some issues have blossomed and deserve some considerations.

It is a noble initiative from authors’ to point out early in the introduction of the article, that in the vast majority of surgery and intensive care services in our country, including large cities, there is not awareness about, nor adoption of intra-abdominal pressure measurement protocols^2^. The protocol proposal based on the guidelines of the World Abdominal Compartment Society^3^ (WSACS) is undoubtedly a necessity nowadays. Despite provision of important foundations by the guideline published in 2013, new literature has emerged in the last seven years.

In the recently published manuscript by Caldas e Ascenção^1^, there is a description that the physical examination is only 50% accurate. However, it is known that the positive predictive value of not diagnosing IAH with physical examination is even greater reaching up to 76%. Physical examination is definitely not reliable, displaying a probability of diagnosis lower than tossing a coin, or 50%. 

The recently published IROI study^4^ demonstrated that IAH occurred in approximately half of all critical patients and was two-fold prevalent in mechanically ventilated patients. The presence of IAH during the observation period significantly increased mortality at 28 and 90 days. The authors also emphasize that the first measurement of intra-abdominal pressure (IAP) is performed at admission to the intensive care unit. They found five variables on the day of admission that were independently associated with the development of IAH^4^. Positive fluid balance, however, was associated with the development of IAH after the first day of hospitalization.

Although IAP can achieve transient values of up to 80mmHg (cough, Valsalva maneuver) physiologically, these values cannot be tolerated for long and constant periods. That said, it is noteworthy that the protocol and flowchart proposed by Caldas and Ascenção does not emphasize that the IAP should be measured serially for a minimum of three consecutive times. Thus, the diagnosis of ACS cannot be completed without high pressures associated with organ dysfunction for three consecutive measurements of six-hour intervals.

Nevertheless, routine IAP measurement is an excellent practice quality marker, since critical patients with measured IAP remain less time in the ICU, even when the diagnosis has extra-abdominal etiology^5^.

The authors use the term IAP continuous measurement, but I fear this is a translation error or a language adaptation from English to Portuguese, since the IAP continuous measurement is impossible. The method for measuring the IAP is standardized as an intra-bladder three-way catheter. Once the patient has a urinary catheter, the bladder is empty; therefore, one has to close the emptying way and instill 25 mL of saline into the bladder to obtain the minimum complacency to move the water column. It is for this simple reason that continuous measurement of IAP is impossible. To be continuous, a minimum of 25 mL of intra-bladder content would be necessary, which is impossible, given the open way to measure urinary output. Throughout the text, the authors assertively recognize the use of a three-way catheter to measure IAP. On the other hand, in Figure 2 of the article, the measurement is performed with a two-way catheter and bladder instillation is performed by puncturing the collector’s portal. This type of approach with a two-way catheter and puncture is contraindicated ^5^ and proscribed by some regional nursing councils, given the increased risk of infection. Furthermore, in the shown flowchart of the manuscript by Caldas and Ascenção (Figure 1) the protocol advises against the patient’s follow-up when the first two measurements are below 12 mmHg, which can be a dangerous advice, since such critical patients may develop IAH during the first week of ICU stay or later ^5^. The most current recommendation is that the measurement of IAP be held as another vital sign in the ICU^5^. One should pay special attention to the possibility of the evolution of IAH/ACS to Polycompartmental Syndrome, a recently understood entity that can certainly compromise the clinical recovery of critically ill patients.

Another important point, more recently described in the literature, is the use of point-of-care ultrasound (POCUS). This new concept is being used to improve diagnostic speed and optimize the treatments of IAH and ACS^6^.

ACS is a potentially lethal condition, caused by any event that produces an increase in IAP and a decrease in abdominal perfusion pressure (APP), inducing ischemia and organ dysfunction. The organization of internal protocols and guidelines based on the WSACS are necessary throughout the national territory, as they positively affect results and patients’ recovery. These also aim to serve as quality markers. The pathophysiological effects are wide-ranging and predispose patients to multiple organ failure if no urgent action is taken.

I leave here my sincere thanks to the authors for the initiative to study the topic and to be concerned with the quality of clinical recovery of their patients.
